# Agrochemicals against Malaria, Sleeping Sickness, Leishmaniasis and Chagas Disease

**DOI:** 10.1371/journal.pntd.0001805

**Published:** 2012-10-25

**Authors:** Matthias Witschel, Matthias Rottmann, Marcel Kaiser, Reto Brun

**Affiliations:** 1 BASF SE, Global Research Herbicides, Ludwigshafen, Germany; 2 Swiss Tropical and Public Health Institute, Basel, Switzerland; 3 University of Basel, Basel, Switzerland; Northeastern University, United States of America

## Abstract

In tropical regions, protozoan parasites can cause severe diseases with malaria, leishmaniasis, sleeping sickness, and Chagas disease standing in the forefront. Many of the drugs currently being used to treat these diseases have been developed more than 50 years ago and can cause severe adverse effects. Above all, resistance to existing drugs is widespread and has become a serious problem threatening the success of control measures. In order to identify new antiprotozoal agents, more than 600 commercial agrochemicals have been tested on the pathogens causing the above mentioned diseases. For all of the pathogens, compounds were identified with similar or even higher activities than the currently used drugs in applied *in vitro* assays. Furthermore, *in vivo* activity was observed for the fungicide/oomyceticide azoxystrobin, and the insecticide hydramethylnon in the *Plasmodium berghei* mouse model, and for the oomyceticide zoxamide in the *Trypanosoma brucei rhodesiense* STIB900 mouse model, respectively.

## Author Summary

Even though agrochemistry and infectious disease control have the same principle goal – the suppression of harmful organisms without harming human health and the environment – there have been only very limited activities to exploit this overlap for the development of new antiinfectious drugs so far. In this study and for the first time, over 600 commercial agrochemicals were systematically screened against the infectious pathogens causing malaria, sleeping sickness, Chagas disease and leishmaniasis. Many highly active compounds with known low mammalian toxicity were identified in cell based assays, and the activity of some of them could even be confirmed in first animal model studies. Further expansion of this concept to other pathogens and the examination of analogues of the identified hits, potentially available from agrochemical companies, would allow for a very efficient source of novel drug candidates.

## Introduction

The Protozoan parasites of the genera *Plasmodium spp.*, *Leishmania spp.*, *Trypanosoma brucei spp.* and *Trypanosoma cruzi*, are the disease causative agents threatening entire populations in mainly resource poor countries around the world.

Malaria, due to infection with *Plasmodium spp.*, is one of the most devastating diseases in developing countries, with 216 million cases in 2010, causing an estimated 655,000 deaths per year [1]. Other recent estimates assume up to 1.2 million deaths per year [2]. For the treatment of malaria several highly active drugs are available, like chloroquine, quinine, mefloquine, atovaquone, artesunate, and their analogs. Thus, malaria is often not included in the list of the *neglected tropical* diseases. Unfortunately, significant resistance to almost all of these drugs has developed; even to the “last resort” artemisinin-derivatives, first cases of delayed clinical efficacy have been reported [3]. Recently, large libraries from pharma companies have been screened against protozoan parasites and some interesting hits [4], [5], [6], [7] have been found, especially against malaria with the spiroindolones currently undergoing clinical evaluation [8], [9].

Most of the promising compounds in the development pipeline are in a rather early clinical stage, so that a high failure rate is expected [10]. Considering the rapid development of resistance, and the challenges seen with the development of malaria vaccines [11], a continuous refilling of research pipelines with compounds in preclinical/clinical evaluation will be necessary, for the long term perspective. Therefore new compounds for resistance management would be highly desirable, even if they might not show the same remarkably high activity levels as the recently promoted peroxide candidates like OZ439 [12]. In addition, the global malaria agenda has shifted from the mere control of clinical cases to malaria elimination and eventually eradication urgently requiring transmission blocking agents [13].

Human African trypanosomiasis (HAT), also known as sleeping sickness, is caused by infections of *T. b. rhodesiense* and *T. b. gambiense*. Populations living in remote rural areas of sub-Saharan Africa are at risk of acquiring HAT. The disease burden in 2000 was estimated at 1.3 Mio DALYs (Disability-Adjusted Life Years) and the estimated number of cases up to 70,000 in 2006 [14]. In recent years the public health situation has improved due to increased monitoring and chemotherapy, resulting in the decrease of reported HAT cases to approximately 10,000 [15]. Only 4 drugs are currently registered as HAT treatment. Pentamidine and suramin are used to treat the hemolymphatic stage (stage 1) of the disease, while melarsoprol and eflornithine (DFMO) are used in stage 2 of the disease when the parasites have invaded the central nervous system (CNS) and which is lethal if untreated. The available drugs are unsatisfactory due to cost, toxicity, poor oral bioavailability, long treatment and lack of efficacy. Melarsoprol is highly toxic, and up to 5% of the second stage patients treated with melarsoprol die of a reactive encephalopathy. Eflornithine treatment is expensive and logistically difficult; it requires four daily intravenous infusions over fourteen days. Recently the eflornithine-nifurtimox combination therapy (NECT) was introduced [16]. The requirement of intravenous administration although reduced to a quarter of injections as compared to monotherapy is still a limitation, with a need for new and more easily administrable drugs.

*Trypanosoma cruzi* infection elicts Chagas disease and is an important public health problem causing approximately 14,000 deaths and 0.7 Mio DALY annually [17]. Treatment options are limited due to toxicity of available drugs, parasite resistance, and poor drug activity during the chronic phase of the disease. Currently there are two medications being used to treat Chagas disease, nifurtimox and benznidazole [18]. Severe toxicity and long treatment requirements are associated with both drugs [19]. Therefore new medications are badly needed for treating this disease especially in its chronic phase.

Leishmaniasis causes approximately 50,000 deaths and 2.1 Mio DALY annually [20]. It threatens about 350 million people around the world and 12 million people are believed to be infected, with 1–2 million estimated new cases every year [21]. Widely used medications are still based on *i.v.* application of antimony compounds like stilbogluconate, resulting in severe side effects. More modern, but also more expensive medications are liposomal amphotericin B, miltefosine, and paromomycin [22].

Thus new affordable and effective therapies are urgently needed to combat these disastrous diseases. Registration requirements for agrochemicals are in some aspects even more stringent than for pharmaceuticals, as side effects that are tolerated for drugs against many life threatening diseases, are not acceptable for agrochemicals that potentially could enter the food chain [23], [24], [25]. As a consequence, all commercialized agrochemicals must go through broad toxicological profiles including e.g. chronic and reprotoxicological studies in different mammalian species, covering at least part of the preclinical studies required for drug development. Furthermore, agrochemicals are highly optimized on agrochemical pest targets with often good selectivities in mammals and excellent temperature and storage stability. Another interesting feature of commercial agrochemicals is the very low production cost of only a few cent/g, as the compounds are produced in highly optimized processes on the multi-ton scale. Surprisingly, these aspects have not led to a systematic evaluation of agrochemicals for pharmacological use so far [26].

Here we present data of over 600 commercial agrochemicals which have been systematically tested for the first time for their antiparasitic activity.

## Materials and Methods

### Chemical library

A library of over 600 compounds (for a list of CAS-numbers and common names of the tested agrochemicals see Supporting Information S1), that are or have been active ingredients in commercial agrochemical products, has been compiled from the BASF compound depository and was dissolved in DMSO stock solutions in a concentration of 10 mg/ml. These samples were then further diluted according to the requirements of the assays. The structural integrity of the dissolved samples has been confirmed subsequently by LCMS-analysis.

### Bioassays

#### *Plasmodium falciparum (Pf)*

*P. falciparum* drug-sensitive strain NF54 was cultivated in a variation of the medium previously described, consisting of RPMI 1640 supplemented with 0.5% ALBUMAX II, 25 mm Hepes, 25 mm NaHCO_3_ (pH 7.3), 0.36 mm hypoxanthine, and 100 µg mL^−1^ neomycin. Human erythrocytes served as host cells. Cultures were maintained in an atmosphere of 3% O_2_, 4% CO_2_, and 93% N_2_ in humidified modular chambers at 37°C. Compounds were dissolved in (CH_3_)_2_SO (10 mg mL^−1^), diluted in hypoxanthine-free culture medium and titrated in duplicates over a 64-fold range in 96-well plates. Infected erythrocytes (1.25% final hematocrit and 0.3% final parasitemia) were added into the wells. After 48 h incubation, 0.5 µCi of [^3^H]hypoxanthine per well was added and the plates were incubated for an additional 24 h. Parasites were harvested onto glass-fiber filters, and radioactivity was counted using a *Betaplate* liquid scintillation counter (*Wallac*, Zurich). The results were recorded and expressed as a percentage of the untreated controls. Fifty percent inhibitory concentrations (IC_50_) were estimated by linear interpolation. Assays were run in duplicate and at least repeated once. Artesunate and chloroquine were used as positive controls.

#### *Trypanosoma brucei rhodesiense (Tb)*

*Trypanosoma brucei rhodesiense* strain STIB900 was isolated in 1982 from a human patient in Tanzania and after several mouse passages cloned and adapted to axenic culture conditions [27] Minimum Essential Medium (50 µL) supplemented with 25 mM HEPES, 1 g L^−1^ additional glucose, 1% MEM non-essential amino acids (100×), 0.2 mM 2-mercaptoethanol, 1 mM Na-pyruvate and 15% heat inactivated horse serum was added to each well of a 96-well microtiter plate. Serial drug dilutions of eleven 3-fold dilution steps covering a range from 100 to 0.002 µg mL^−1^ were prepared. Then 4×10^3^ bloodstream forms of *T. b. rhodesiense* STIB 900 in 50 µL was added to each well and the plate incubated at 37°C under a 5% CO_2_ atmosphere for 70 h. 10 µL resazurin solution (resazurin, 12.5 mg in 100 ml double-distilled water) was then added to each well and incubation continued for a further 2–4 h [28]. Then the plates were read with a Spectramax Gemini XS microplate fluorometer (Molecular Devices Cooperation, Sunnyvale, CA, USA) using an excitation wave length of 536 nm and an emission wave length of 588 nm. The IC_50_ values were calculated by linear regression [29] from the sigmoidal dose inhibition curves. Melarsoprol was used as positive control.

#### *Trypanosoma cruzi (Tc)*

Rat skeletal myoblasts (L-6 cells) were seeded in 96-well microtiter plates at 2000 cells/well in 100 µL RPMI 1640 medium with 10% FBS and 2 mm L-glutamine. After 24 h, the medium was replaced by 100 µL per well medium containing 5000 trypomastigote forms of *T. cruzi* Tulahuen strain C2C4 containing the β-galactosidase (Lac Z) gene (Buckner et al. 1996) [30]. After 48 h the medium was removed from the wells and replaced by 100 µL fresh medium with or without a serial drug dilution of eleven 3-fold dilution steps covering a range from 100 to 0.002 µg mL^−1^. After 96 h of incubation, the plates were inspected under an inverted microscope to assure growth of the controls and sterility. Then the substrate CPRG/Nonidet (50 µL) was added to all wells. A color reaction developed within 2–6 h and could be measured photometrically at 540 nm with a VersaMax microplate reader (Molecular Devices Cooperation, Sunnyvale, CA, USA). The IC_50_ values were calculated by linear regression from the sigmoidal dose inhibition curves. Benznidazole was used as positive control.

#### *Leishmania donovani (Ld)*

Amastigotes of *L. donovani* strain MHOM/ET/67/L82 were grown in axenic culture at 37°C in SM medium [31] at pH 5.4 supplemented with 10% heat-inactivated fetal bovine serum under an atmosphere of 5% CO_2_ in air. One hundred microlitres of culture medium with 10^5^ amastigotes from axenic culture with or without a serial drug dilution were seeded in 96-well microtitre plates. Serial drug dilutions of eleven 3-fold dilution steps covering a range from 100 to 0.002 µg mL^−1^ were prepared. After 70 h of incubation the plates were inspected under an inverted microscope to assure growth of the controls and sterile conditions. 10 µL resazurin solution (resazurin, 12.5 mg in 100 ml double-distilled water) [32] were then added to each well and the plates incubated for another 2 h. Then the plates were read with a Spectramax Gemini XS microplate fluorometer (Molecular Devices Cooperation, Sunnyvale, CA, USA) using an excitation wave length of 536 nm and an emission wave length of 588 nm. The IC_50_ values were calculated by linear regression from the sigmoidal dose inhibition curves. Miltefosine was used as positive control.

#### *P. berghei in vivo* model

From a donor mouse with approximately 30% parasitaemia (PbANKA-GFP_CON_) [33], heparinized blood (containing 50 µL of 200 u mL^−1^ Heparin) was taken and diluted in physiological saline to 10^8^ parasitized erythrocytes per mL. Of this suspension, 0.2 mL were injected intravenously (*i.v.*) into experimental groups of 3 mice, and a control group of 5 mice. 6, 24, 48 and 72 hours after infection (6 hour time point omitted during 3 times treatment), the experimental groups were treated with a single daily dose (*p.o.* or *s.c.*). 24 hours after the last drug treatment (96 hours after infection), 1 µL tail blood was taken and the parasitaemia determined with a FACScan. The difference between the mean value of the control group and those of the experimental groups was calculated and expressed as a percent relative to the control group ( = activity). The survival of the animals was monitored up to 30 days. Mice surviving for 30 days were checked for parasitaemia by slide reading. A compound was considered curative if the animal survived to day 30 post-infection with no detectable parasites. All protocols and procedures were reviewed and approved by the local veterinary authorities of the Canton Basel-Stadt.

#### *T. b. rhodesiense in vivo* model

The STIB900 acute mouse model mimics the first stage of the disease [34], [35]. Four female NMRI mice were used per experimental group. Each mouse was inoculated i.p. with 10^4^ bloodstream forms of STIB900. Heparinized blood from a donor mouse with approximately 5×10^6^ mL^−1^ parasitaemia was suspended in PSG to obtain a trypanosome suspension of 4×10^4^ mL^−1^. Each mouse was injected with 0.25 ml. Compound treatment was initiated 3 days post-infection on four consecutive days for all administration routes (i.p., p.o.) in a volume of 10 mL kg^−1^. Three mice served as infected-untreated controls. They were not injected with the vehicle alone since we have established in our labs that these vehicles do not affect parasitaemia nor the mice. Parasitaemia was monitored using smears of tail-snip blood twice a week after treatment for two weeks followed by once a week until 60 days post-infection. Mice were considered cured when there was no parasitaemia relapse detected in the tail blood over the 60-day observation period. Mean relapse days were determined as day of relapse post-infection of mice. All protocols and procedures were reviewed and approved by the local veterinary authorities of the Canton Basel-Stadt.

### Ethics statement

All work was conducted in accordance to relevant national and international guidelines. The *in vivo* efficacy studies were approved by the veterinary authorities of the Canton Basel-Stadt. The in vivo studies were carried out under license No. 1731 and license No. 739 of the Kantonales Veterinäramt, CH-4025 Basel, Switzerland adhering to the Tierschutzverordnung from 23.04.2008 (based on the Tierschutzgesetz from 26.12.2005).

## Results and Discussion

Starting with the analysis of the phylogenetic relationship of the pests combated with agrochemicals, and the most important tropical infectious disease pathogens as defined by WHO [36], the close relationship of oomycetes, to which important agricultural pathogens like potato blight or downy mildew belong, with protozoan parasites was realized [37]. As a result, a first set of oomyceticidal agrochemicals was tested, resulting in a number of interesting hits. Based on this finding, over 600 commercially available agrochemicals were selected and their activity against the tropical disease pathogens *Plasmodium falciparum*, *Leishmania donovani*, *Trypanosoma cruzi* and *Trypanosoma brucei rhodensiense* tested in cell based screens.

### Activity against *Plasmodium falciparum*

#### *In vitro* activity against *Plasmodium falciparum*

**Figure 1:**
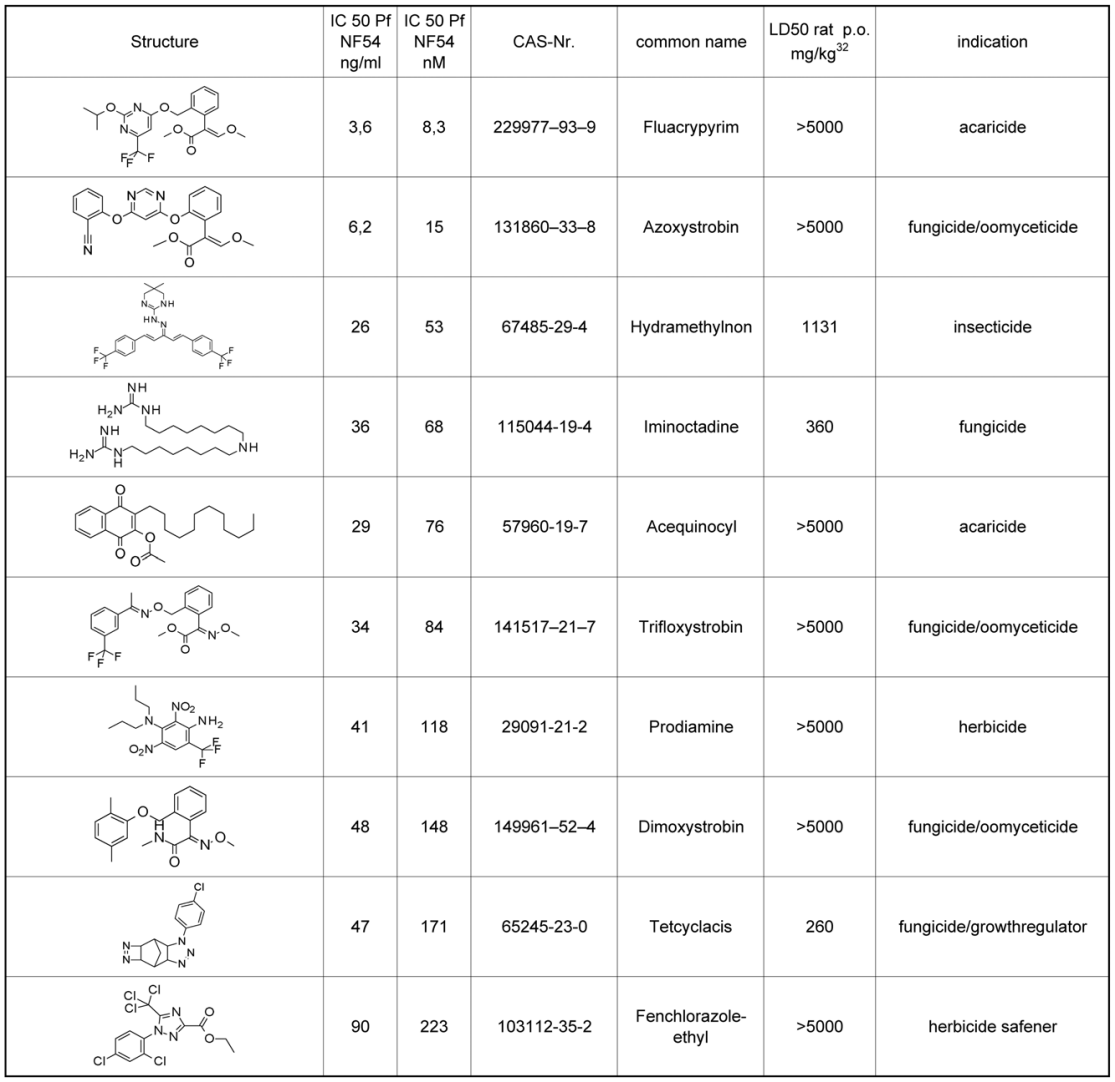
*In vitro* activity of the top 10 most active commercial agrochemicals on *P. falciparum* NF54 strain. The IC_50_ values are the means of two independent assays; the individual values vary by less than a factor of 2.

For 24 commercial agrochemicals sub-µM activity on *P. falciparum* could be shown (Figure 1), therefore only the most active compounds will be discussed in more detail. The standards Artesunate (LD_50_ rat i.p. 352 mg/kg; LD_50_ p.o. not available) [38], [39] and Chloroquine in the same assay (LD_50_ rat p.o. 330 mg/kg) [40] exhibited an activity of 5.7 and 17.1 nM, respectively.

Fluacrypyrim, (LD_50_ rat p.o. >2000 mg/kg) [41] demonstrated the best activity against *P. falciparum* of all agrochemicals with an IC_50_ of 8.3 nM. Fluacrypyrim is an acaricide from the group known as the strobilurins, which is mainly used in Japan against mites in orchards. The acaricidal mode of action is the inhibition of respiration by binding to the Qo-site of the bc1-complex [42]. This target is also addressed by the antimalarial drug Atovaquone. Other strobilurin-analogues have been examined before as antimalarials [43].

Azoxystrobin (LD_50_ rat p.o. >5000 mg/kg), also a strobilurin, showed activity at 15 nM. Azoxystrobin is a broadspectrum fungicide and oomyceticide with annual sales of >1 bn€, and production volumes of several 1000 tons/year. It is one of the predominant agrochemicals in the market. Azoxystrobin has also been identified in a high throughput screening campaign of Glaxo-SmithKline, where it showed an IC_50_ value of 41 nM against *P. falciparu*m [44]. This result has surprisingly not been mentioned in the analysis and no follow up has been published.

Hydramethylnon (LD_50_ rat p.o. 1131 mg/kg), an insecticide used in baits against ants, termites and cockroaches, showed 53 nM activity. It is also inhibiting the respiration chain, but probably not at the Qo binding site [45].

Iminoctadine (LD_50_ rat p.o. 300 mg/kg), a broad spectrum fungicide, showed 68 nM activity, with the mode of action presumed to be interaction with cell membranes and lipid biosynthesis. Related bisguanidines have also been examined extensively as antiprotozoal drugs before [46].

Acequinocyl (LD_50_ rat p.o. >5000 mg/kg), an acaricide used predominantly against mites in ornamentals, exhibited an IC_50_ value of 76 nM. It is also inhibiting the Qo-site in the bc1-complex like atovaquone, to which it also shows some structural similarities.

Additional strobilurins with broadspectrum fugicidal and oomyceticidal activity were tested including trifloxystrobin (LD_50_ rat p.o. >5000 mg/kg), dimoxystrobin (LD_50_ rat p.o. >5000 mg/kg), picoxystrobin (LD_50_ rat p.o. >5000 mg/kg), and pyraoxystrobin (LD_50_ not available), resulting in IC_50_ values of 84, 148, 305 and 859 nM activity, respectively.

The pre-emergence herbicides from the dinitroaniline-type including prodiamine (LD_50_ rat p.o. >5000 mg/kg), dinitramine (LD_50_ rat p.o. 3000 mg/kg), and fluchloralin (LD_50_ rat p.o. 1550 mg/kg), showed values of 118, 253 and 816 nM activity, respectively. Their mode of action is the inhibition of mitosis. Other herbicidal dinitroanilines have been shown before to have antiplasmodial activity, but on a significantly weaker level [47].

The plant growth regulator tetcyclacis (LD_50_ rat p.o. 261 mg/kg) inhibits P450 enzymes [48] and exhibits an IC_50_ value of 194 nM.

Fenchlorazol-ethyl (LD_50_ rat p.o. >5000 mg/kg), an herbicide safener used in cereals, showed 223 nM activity. Furthermore, the corresponding acid, which is potentially the first metabolite of fenchlorazol-ethyl, showed no activity in the assay. Other sub-µM agrochemicals are fluazinam (IC_50_ = 258 nM), cafenstrole (493 nM), difenthiuron (560 nM), fenamidone (641 nM) and butamifos (816 nM).

The biocides fentin acetate (33 nM) (LD_50_ rat p.o. 140–278 mg/kg), berberine (83 nM) (LD_50_ rat i.v. 60 mg/kg), cycloheximide (101 nM) (LD_50_ rat p.o. 2 mg/kg), fentin hydroxide (408 nM) (LD_50_ rat p.o. 150–165 mg/kg) and thiocyclam (525 nM) (LD_50_ rat p.o. 370 mg/kg) which are used in agrochemistry e.g. as seed dressing, also showed high activity against *P. falciparum*, but were not further followed up due to their published high toxicity in mammalian species.

#### *In vivo* antimalarial activity

In the *P. berghei* mouse model azoxystrobin showed after 4×100 mg/kg p.o. application no significant activity using the Tween-formulated a.i.; but using the aqueous suspension of the commercial fungicidal formulation (200 g/l suspension concentrate) in p.o. application, an extension of survival time from 6–7 to 10.7 days compared to untreated control animals was achieved. With s.c. application of the aqueous formulation a reduction of parasitemia by 98% compared to the untreated mice 24 hrs after last compound application (or 96 hrs after infection) and an extension of the survival time from 6–7 to 13.3 days was observed. This suggests some potential for further optimization of the delivery system.

Hydramethylnon showed with 4×100 mg/kg s.c. application a reduction of parasitemia of 87% and an extension of survival time from 6–7 to 14 days. Furthermore, with a 4×100 mg/kg p.o. application, the parasitemia was reduced by 96% and the survival time was increased to up to 16 days. Considering the challenging physicochemical properties, moderate transfer factor and the non-optimized dosing regime and formulation of hydramethylnon there might still be some potential to reach a sufficient activity level especially in combination therapies. This warrants further follow up and is currently under examination.

### Activity on *Trypanosoma cruzi* (Chagas disease)

**Figure 2:**
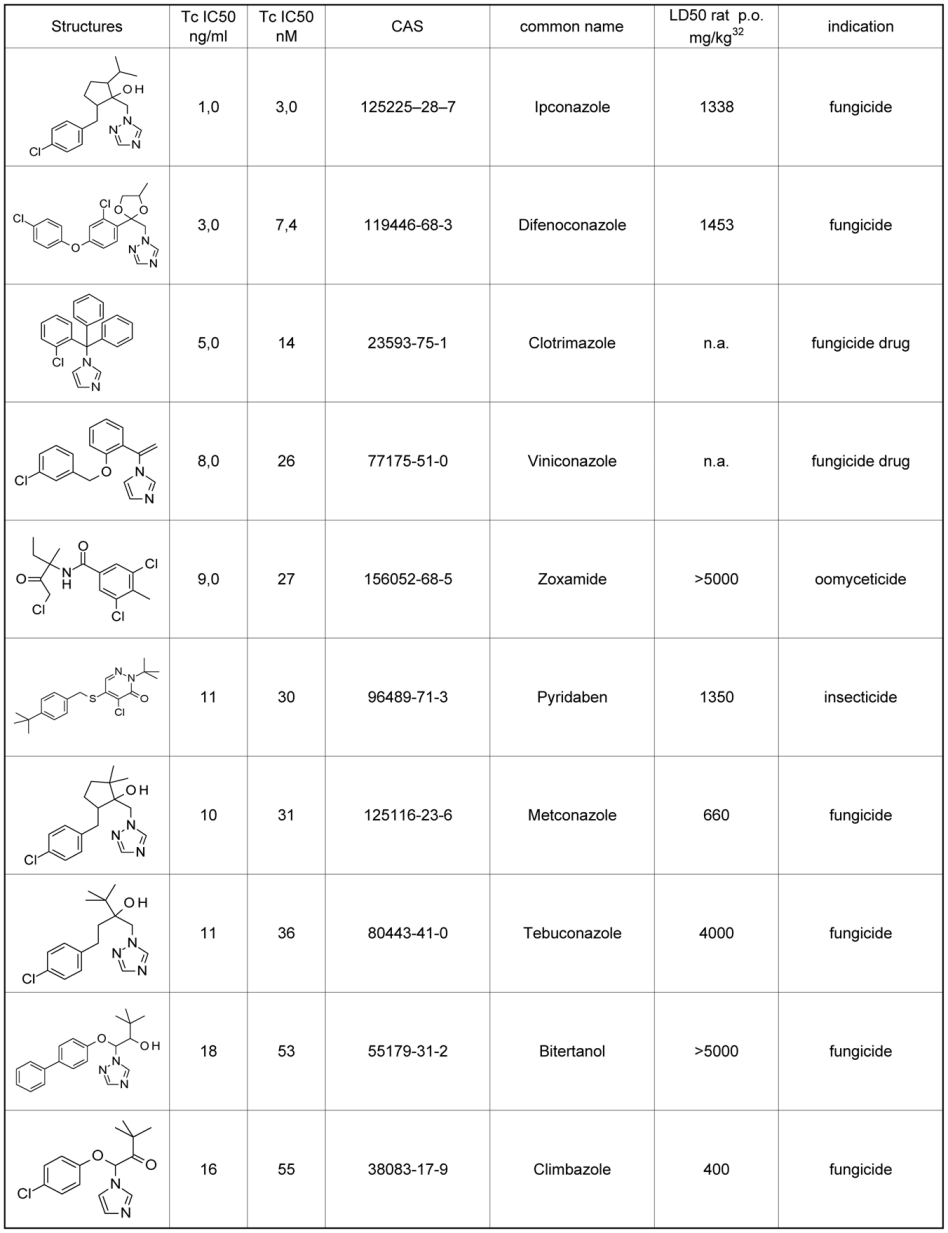
Top 10 most active commercial agrochemicals on *T. cruzi*. The IC_50_ values are the means of two independent assays; the individual values vary by less than a factor of 2.

38 agrochemicals with sub-µM activity on *T. cruzi* were identified, many of which being azoles with P450-inhibiting activity (Figure 2). P450-monoxygenases have been discussed before as targets against *T. cruzi*, especially the sterol 14α-demethylase [49].

The standard drug benznidazole (LD_50_ rat p.o. not available) [50], [51] has an IC_50_ of 1871 nM in this assay.

Ipconazole (LD_50_ rat p.o. 888 mg/kg), has an IC_50_ of 3.0 nM, the most active agrochemical against *T. cruzi*. It is a fungicide used predominantly in seed dressing. The tested material is, like the commercial material, racemic and a mixture of diastereomers, therefore an enantiopure isomer could potentially have even higher activity.

Difenoconazole (LD_50_ rat p.o. 1453 mg/kg), a broad spectrum and systemic fungicide, showed an IC_50_ value of 7.4 nM. This commercial agrochemical is again a racemic diasteromeric mixture and could therefore also have intrinsically higher activity as a pure isomer.

Clotrimazole (14 nM), and viniconazole [52] (26 nM), are two azole drugs used against fungal skin infections, that have also been discussed as agro fungicides and therefore have been tested in this screen. As they have a complete pharmacological dossier they might also be interesting drug candidates.

Zoxamide (LD_50_ rat p.o. >5000 mg/kg), a broadspectrum oomyceticide used in fruits and vegetables, showed 27 nM activity. It is sold and was tested as a racemate. Its mode of action against oomycetes is the inhibition of microtubule formation.

Pyridaben (30 nM), and tolfenpyrad (55 nM), are insecticides/acaricides inhibiting the complex 1 in the mitochondrial electron transport chain.

A number of further azole fungicides showed activities below 100 nM including metconazole 31 nM, tebuconazole 36 nM, bitertanol 35 nM, climbazole 55 nM, prochloraz 69 nM, hexaconazole 73 nM, and fenapanil 99 nM. Further agrochemicals with high activity in this assay were penconazole (130 nM), epoxyconazole (136 nM), imazalil (148 nM), propiconazole (160 nM), fenarimol (193 nM), fluquinconazole (199 nM), picoxystrobin (248 nM), cyproconazole (257 nM), myclobutanil (374 nM), tetraconazole (478 nM), and pyrifenox (491 nM).

In spite of the excellent *in vitro* activity initial experiments in a *T. cruzi* mouse model did so far not show *in vivo* efficacy for selected hits (personal communication Nazaré Soiro).

### Activity on *Leishmania donovani* (Leishmaniasis)

**Figure 3:**
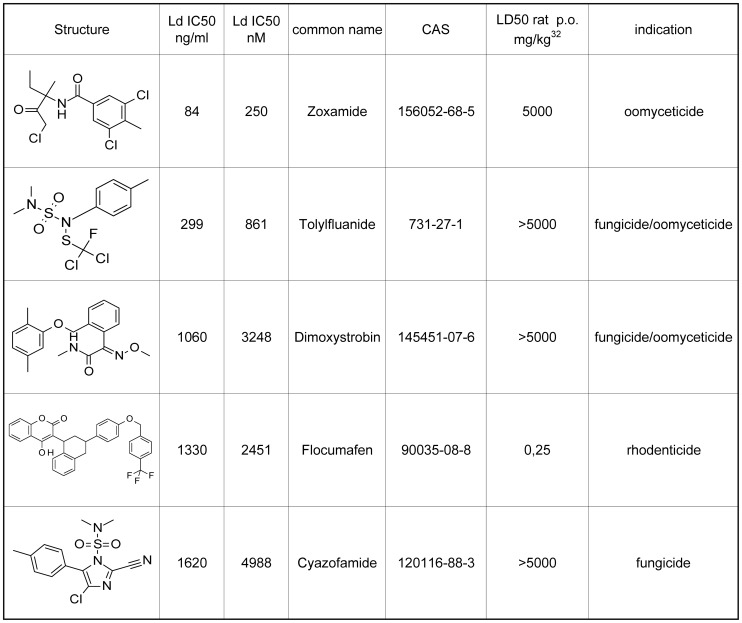
Most active commercial agrochemicals on *L. donovani*. The IC_50_ values are the means of two independent assays; the individual values vary by less than a factor of 2.

Against *L. donovani* only two agrochemicals showed sub-µM activity (Figure 3). The standard miltefosine (LD_50_ rat p.o. 246 mg/kg) showed in this assay an IC_50_ value of 250 nM.

Zoxamide (LD_50_ rat p.o. >5000 mg/kg) showed an IC_50_ of 250 nM. The oomyceticidal compound has been discussed in the *T. cruzi* section.

Tolylfluanid (LD_50_ rat p.o. >5000 mg/kg) resulted in an IC_50_ value of 861 nM. It is a protective fungicide and oomyceticide with presumed thiol conjugating activity.

Other agrochemicals with moderate activity against *L. donovani* were flocumafen (2451 nM), dimoxystrobin (3248 nM), bromofenoxin (3839 nM), cyhexatin (4517 nM), and cyazofamid (4988 nM).

### Activity on *Trypanosoma brucei rhodensiense*

#### *In vitro* activity against *T. b. rhodensiense*

The standard melarsoprol, an arsenate derivative (LD_50_ in mouse *i.v.* 44 mg/kg), showed an IC_50_ value of 5 nM in this assay.

**Figure 4:**
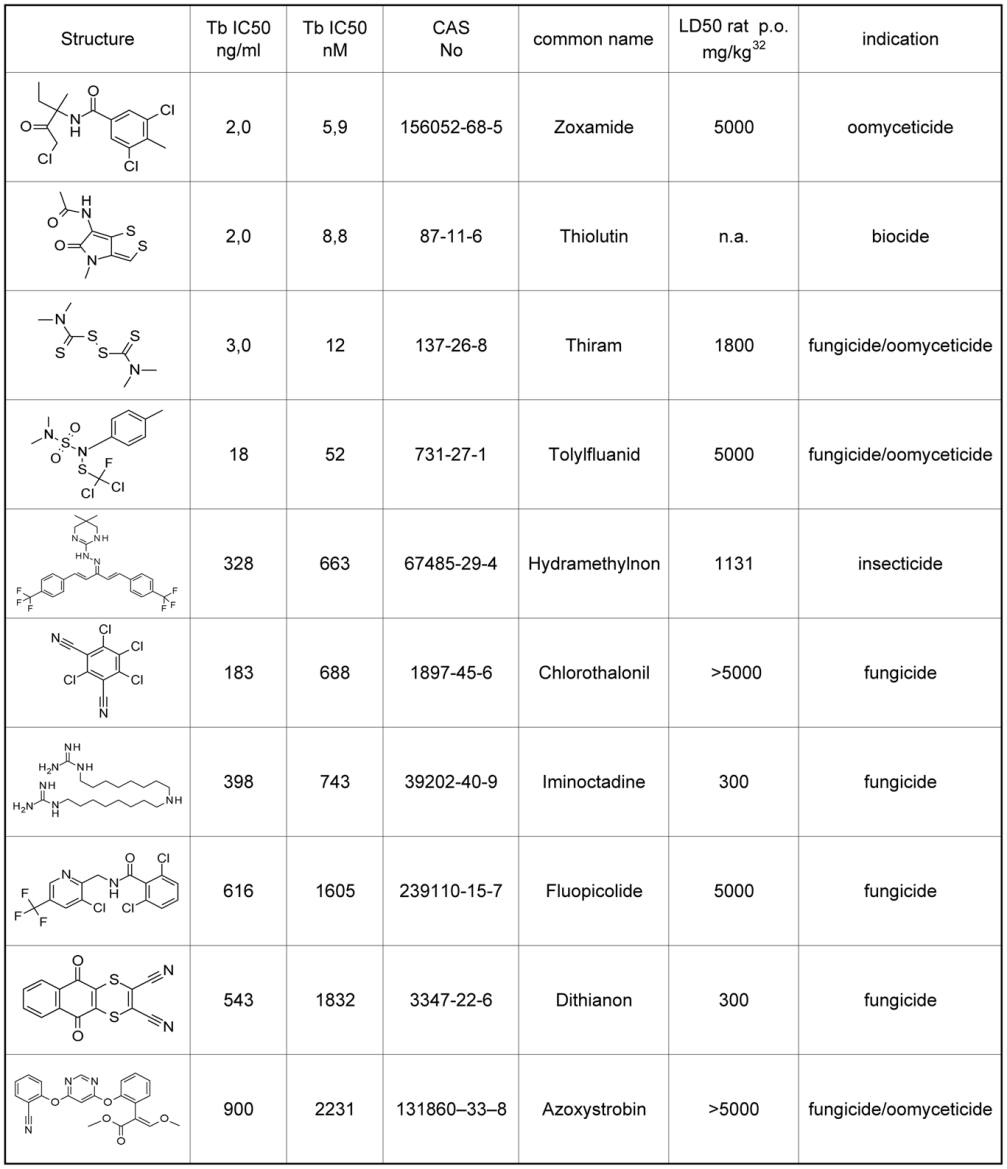
Top 10 most active commercial agrochemicals on *T. b. rhodesiense*. The IC_50_ values are the means of two independent assays; the individual values vary by less than a factor of 2.

Seven sub-µM active agrochemicals could be identified in the *T. b. rhodensiense* assay (Figure 4). The two agrochemicals thiram (IC_50_ 12 nM), and thiolutin (IC_50_ 9 nM) [53] are known to have rather high cytotoxicity in cell systems, which likely interferes with this assay.

Zoxamide (LD_50_ rat p.o. >5000 mg/kg) showed the highest activity with an IC_50_ value of 6 nM. This oomyceticidal compound has been discussed above. Toylfluanid (LD_50_ rat p.o. >5000 mg/kg), showed an IC_50_ value of 52 nM, in addition to its activity against *L. donovani*.

In addition to the above described antimalarial activity, hydramethylnon (LD_50_ rat p.o. 1131 mg/kg), showed 663 nM activity.

Chlorothalonil (LD_50_ rat p.o. >5000 mg/kg), a protective fungicide with thiol conjugating activity, showed 688 nM activity.

Iminoctadin/guacetin (LD_50_ rat p.o. 360 mg/kg), showed 743 nM activity and is discussed in the *P. falciparum* chapter.

#### *In vivo* activity against *T. b. rhodensiense*

Zoxamide has been tested in the *T. b. rhodesiense* mouse model for the acute phase of human African trypanosomiasis. Zoxamide showed with 4×200 mg/kg i.p. a weak activity. On day 7 post infection, 24 hours after the last treatment, no *T. b. rhodesiense* could be detected; on day 10 all mice showed a relapse.

### Conclusion

Due to the split of most life science companies into their agro- and pharma branches in the 1990s, the companies active in agrochemistry have not been involved in the recent screening activities to identify new drugs against infectious tropical diseases, even though agrochemicals might have a high potential to yield interesting hits for these applications.

In this cooperation between industrial and public partners, it was shown for several commercial agrochemicals that they are highly active against some of the most important pathogens of infectious tropical diseases. Interestingly as anticipated, several of the oomyceticides (strobilurins against *P. falciparum*, zoxamide against *T. b. rhodesiense* and *L. donovani*) were active against these protozoans, but also other agrochemicals (e.g. hydramethylnon against *P. falciparum*; azoles like iproconazole against *T. cruzi*) showed very interesting activities. Exemplified by one of the major commercial agrochemicals, the fungicide azoxystrobin, as well as for the insecticide hydramethylnone, the reduction of parasitemia, and significant life extension for *P. berghei* infected mice was achieved. For zoxamide, an effect against *T. brucei* in the mouse model was also demonstrated. This successful *in vitro– in vivo* transfer without galenic optimization could not be taken for granted, as these agrochemicals have not been optimized for mammalian pharmacokinetics.

There is still a high probability that the identified hits in the end might not be suitable for human use, as there are still several hurdles to overcome. However, the results of this highly focussed and relatively low input approach are more promising than could have been hoped for. It is especially noteworthy, that the screen of less than 700 agrochemical resulted in e.g. 24 new sub-µM hits against *P. falciparum*, compared to 4 new sub-µM hit in over 2687 recently tested commercial drugs (excluding known antimicrobial and anti-cancer a.i.) [54], [55]. This clearly demonstrates that agrochemistry can be a very interesting and so far untapped source of new leads, and maybe even drug candidates, against protozoal diseases.

It would also be very interesting to screen commercial agrochemicals against the pathogens of other neglected diseases, like schistosomes, nematodes, food borne trematodes, diarrhoeal amoebas and also tropical bacterial pathogens, for which good antibiotic cures are missing. These studies are still to be done.

## Supporting Information

Supporting Information S1 **CAS-numbers and common names of the tested agrochemicals.**
(DOC)
