# Supplementary material for: Agrochemicals against Malaria, Sleeping Sickness, Leishmaniasis and Chagas Disease
Source: PLoS Negl Trop Dis. 2012 Oct 25;6(10):e1805. doi: 10.1371/journal.pntd.0001805 (PMC3493374; doi:10.1371/journal.pntd.0001805)
Supplement: Supporting Information S1 — CAS-numbers and common names of the tested agrochemicals. (DOC) [file pntd.0001805.s001.doc]

Names and CAS-Numbers of all tested agrochemicals:

| Product Name | CAS No |
| --- | --- |
|  |  |
| AZIPROTRYNE | 4658-28-0 |
| ISOLANE | 119-38-0 |
| SERAPHOS | 31218-83-4 |
| TEBUFENOZIDE | 112410-23-8 |
| (7E,9Z)-DODECADIENYL ACETATE | 54364-62-4 |
| (E)-5-DECEN-1-YL ACETATE | 38421-90-8 |
| (E)-8-DODECENYL ACETATE | 37338-40-2 |
| 1H-INDOLE-3-ACETIC ACID | 87-51-4 |
| 1-NAPHTHYLACETIC ACID | 86-87-3 |
| 2-(P-CHLOROPHENOXY)-2-METHYLPROPIONIC ACID | 882-09-7 |
| 2,3,5-TRIIODOBENZOIC ACID | 88-82-4 |
| 2,4,5-T | 93-76-5 |
| 2,4,5-T-BUTYLESTER | 93-79-8 |
| 2,4,5-T-METHYL-ESTER | 4841-20-7 |
| 2,4,5-TRICHLOROPHENOXY-METHYLACETATE | 1928-37-6 |
| 2,4-D | 94-75-7 |
| 2,4-D-(2-ETHYLHEXYL)-ESTER | 1928-43-4 |
| 2,4-DECADIEN-CARBONSAEUREMETHYLESTER | 53172-59-1 |
| 2,4-D-METHYLESTER | 1928-38-7 |
| 2-ISOVALERYL-1,3-INDANDIONE | 83-26-1 |
| 3-(2-PYRIDYL)-4H-1,2,4-TRIAZIN-5-ONE | 49772-17-0 |
| 3-(P-CHLOROPHENYL)-1,1-DIMETHYLUREA | 150-68-5 |
| 3,4-D | 588-22-7 |
| 3-INDOLEBUTYRIC ACID | 133-32-4 |
| 4-(4-(ACETYLOXY)-PHENYL)-2-BUTANON | 3572-06-3 |
| 4,4-DIMETHYL-MORPHOLINIUM CHLORIDE | 23165-19-7 |
| 4-CPP | 3307-39-9 |
| 8-CHINOLINOL | 148-24-3 |
| ABG-3168 | 55720-26-8 |
| ACARALATE | 5836-10-2 |
| ACEPHATE | 30560-19-1 |
| ACEQUINOCYL | 57960-19-7 |
| ACEQUINOCYL | 57960-19-7 |
| ACETAMIPRID | 135410-20-7 |
| ACETOCHLOR | 34256-82-1 |
| ACETOPROLE | 209861-58-5 |
| ACIFLUORFEN | 50594-66-6 |
| ACIFLUORFEN-SODIUM | 62476-59-9 |
| ACLONIFEN | 74070-46-5 |
| ACTELLIC | 29232-93-7 |
| ALACHLOR | 15972-60-8 |
| ALBENDAZOLE | 54965-21-8 |
| ALLETHRIN | 584-79-2 |
| ALLOXYDIM | 55634-91-8 |
| ALPHAMETHRIN | 52315-07-8 |
| AMETOCTRADIN | 865318-97-4 |
| AMETRYNE | 834-12-8 |
| AMICARBAZONE | 129909-90-6 |
| AMIDOFLUMET | 84466-05-7 |
| AMIDOSULFURON | 120923-37-7 |
| AMINOETHOXYVINYLGLYCIN | 49669-74-1 |
| AMINOPYRALID | 150114-71-9 |
| AMINOZID | 1596-84-5 |
| AMITRAZ | 33089-61-1 |
| AMITROLE | 61-82-5 |
| ANILAZIN | 101-05-3 |
| ANILOPHOS ANILOGUARD | 64249-01-0 |
| ANTHRAQUINONE | 84-65-1 |
| ASULAM | 3337-71-1 |
| ATRAZIN | 1912-24-9 |
| AZACONAZOLE | 60207-31-0 |
| AZAFENIDIN | 68049-83-2 |
| AZINPHOS-METHYL | 86-50-0 |
| AZOXYSTROBIN | 131860-33-8 |
| BARBAN | 101-27-9 |
| BASAMAIZE | 21267-72-1 |
| BAYTHION | 14816-18-3 |
| BEFLUBUTAMIDE | 113614–08–7 |
| BENALAXYL | 71626-11-4 |
| BENAZOLIN | 3813-05-6 |
| BENFLURALIN | 1861-40-1 |
| BENODANIL | 15310-01-7 |
| BENOMYL | 17804-35-2 |
| BENOXACOR | 98730-04-2 |
| BENSULFURON-METHYL | 83055-99-6 |
| BENSULIDE | 741-58-2 |
| BENTAZONE | 25057-89-0 |
| BENTRANIL | 1022-46-4 |
| BENZADOX | 5251-93-4 |
| BENZFENDIZONE | 158755-95-4 |
| BENZOBICYCLON | 156963-66-5 |
| BENZTHIAZURON | 1929-88-0 |
| BENZYLAMINOPURIN | 1214-39-7 |
| BERBERINE HYDROCHLORIDE | 633-65-8 |
| BETA-NAPHTHOXYACETIC ACID | 120-23-0 |
| BIALAPHOS | 35597-43-4 |
| BIDIPHEN | 97-18-7 |
| BIFENAZATE | 149877-41-8 |
| BIFENOX | 42576-02-3 |
| BINAPACRYL | 485-31-4 |
| BION | 135158-54-2 |
| BIPHENTHRIN | 82657-04-3 |
| BISPYRIBAC | 125401-75-4 |
| BISPYRIBAC-SODIUM | 125401-92-5 |
| BITERTANOL | 55179-31-2 |
| BIXAFEN | 581809-46-3 |
| BLASTICIDIN | 2079-00-7 |
| BOSCALID | 188425-85-6 |
| BROMAZIL | 33586-66-2 |
| BROMOBUTIDE | 74712-19-9 |
| BROMOFENOXIM | 13181-17-4 |
| BROMOPHOS | 4824-78-6 |
| BROMOXYNIL | 1689-84-5 |
| BROMPYRAZON | 3042-84-0 |
| BROMUCONAZOLE | 116255-48-2 |
| BROMURON | 3408-97-7 |
| BRYOKININ | 2365-40-4 |
| BUCTRIL | 1689-99-2 |
| BUPIRIMATE | 41483-43-6 |
| BUPROFEZIN | 69327-76-0 |
| BUPROFEZIN | 69327-76-0 |
| BUSAN | 21564-17-0 |
| BUTACHLOR | 23184-66-9 |
| BUTOPYRONOXYL | 532-34-3 |
| BUTRALIN | 33629-47-9 |
| BUTROXYDIM | 138164-12-2 |
| BUTURON | 3766-60-7 |
| BUTYLATE | 2008-41-5 |
| BUTYRYLPHENOL | 2887-61-8 |
| CAFENSTROLE | 125306-83-4 |
| CALOXYDIM | 149979-41-9 |
| CAPTAFOL | 2425-06-1 |
| CAPTAN | 133-06-2 |
| CARBARYL | 63-25-2 |
| CARBENDAZIM | 10605-21-7 |
| CARBETAMID | 16118-49-3 |
| CARBOFURAN | 1563-66-2 |
| CARBOSULFAN | 55285-14-8 |
| CARBOXIN | 5234-68-4 |
| CARFENTRAZONE-ETHYL | 128639-02-1 |
| CARPROPAMID | Formularbeginn  104030-54-8 Formularende |
| CDEA | 2315-36-8 |
| CHINOMETHIONAT | 2439-01-2 |
| CHLOMETHOXYFEN | 32861-85-1 |
| CHLORALOSE | 15879-93-3 |
| CHLORAMBEN | 133-90-4 |
| CHLORAMPHENICOL | 56-75-7 |
| CHLORANIL | 118-75-2 |
| CHLORBENSIDE | 103-17-3 |
| CHLORBENZILAT | 510-15-6 |
| CHLORBROMURON | 13360-45-7 |
| CHLORBUFAM | 1967-16-4 |
| CHLORDIMEFORM | 19750-95-9 |
| CHLORDIMEFORM | 6164-98-3 |
| CHLORFENAC | 85-34-7 |
| CHLORFENAPYR | 122453-73-0 |
| CHLORFENPROP-METHYL | 14437-17-3 |
| CHLORFLUAZURON | 71422-67-8 |
| CHLORIDAZON | 1698-60-8 |
| CHLORMEQUAT CHLORIDE | 999-81-5 |
| CHLOROFLURAZOLE | 3615-21-2 |
| CHLOROTOLURON | 15545-48-9 |
| CHLOROXURON | 1982-47-4 |
| CHLORPHOXIME | 14816-20-7 |
| CHLORPROPHAM | 101-21-3 |
| CHLORPYRIFOS | 2921-88-2 |
| CHLORSULFURON | 64902-72-3 |
| CHLORTHAL-DIMETHYL | 1861-32-1 |
| CHLORTHALONIL | 1897-45-6 |
| CHLOZOLINATE | 84332-86-5 |
| CHOLINE CHLORIDE | 67-48-1 |
| CINIDON-ETHYL | 142891-20-1 |
| CLETHODIM | 99129-21-2 |
| CLIMBAZOLE | 38083-17-9 |
| CLOETHOCARB | 51487-69-5 |
| CLOFENTEZINE | 74115-24-5 |
| CLOMAZONE | 81777-89-1 |
| CLOPROP | 101-10-0 |
| CLOPROXYDIM | 95480-33-4 |
| CLOQUINTOCET-MEXYL | 99607-70-2 |
| CLOTHIANIDIN | 210880-92-5 |
| CLOTRIMAZOL | 23593-75-1 |
| CLOTRIMAZOLE | 23593-75-1 |
| CLOXYFONAC | 6386-63-6 |
| CUMYLURON | 99485-76-4 |
| CYANAZINE | 21725-46-2 |
| CYANOPHOS | 2636-26-2 |
| CYAZOFAMID | 120116-88-3 |
| CYAZYPYR | 736994-63-1 |
| CYCLOATE | 1134-23-2 |
| CYCLOHEXIMID | 66-81-9 |
| CYCLOSULFAMURON | 136849-15-5 |
| CYCLOXYDIM | 101205-02-1 |
| CYCLURON | 2163-69-1 |
| CYFLUFENAMID | 180409-60-3 |
| CYHEXATIN | 13121-70-5 |
| CYMOXANIL | 57966-95-7 |
| CYMOXANIL | 57966-95-7 |
| CYPROCONAZOLE | 94361-06-5 |
| CYPRODINIL | 121552-61-2 |
| CYPROFURAM | 69581-33-5 |
| CYPROSULFAMIDE | 221667-31-8 |
| DALAPON | 75-99-0 |
| DDT | 50-29-3 |
| DEHYDROACETIC ACID | 520-45-6 |
| DELTAMETHRIN | 52918-63-5 |
| DIAFENTHIURON | 80060-09-9 |
| DIAFENTHIURON | 80060-09-9 |
| DIALLATE | 2303-16-4 |
| DIAZINON | 333-41-5 |
| DICAMBA | 1918-00-9 |
| DICAPTHON | 2463-84-5 |
| DICHLOBENIL | 1194-65-6 |
| DICHLOBUTRAZOL | 66345-62-8 |
| DICHLOFLUANID | 1085-98-9 |
| DICHLONE | 117-80-6 |
| DICHLOPROP-METHYL-ESTER | 57153-17-0 |
| DICHLORAN | 99-30-9 |
| DICHLOROPHEN | 97-23-4 |
| DICHLOROPHENYLSUCCINIMIDE | 24096-53-5 |
| DICHLORPHOS | 62-73-7 |
| DICHLORPROP | 120-36-5 |
| DICHLORPROP-2-ETHYLESTER | 79270-78-3 |
| DICHLOZOLINE | 24201-58-9 |
| DICLOFOP-METHYL | 51338-27-3 |
| DICOFOL | 115-32-2 |
| DICRYL | 2164-09-2 |
| DICUMAROL | 66-76-2 |
| DIDION | 82-66-6 |
| DIETHATYL-ETHYL | 38727-55-8 |
| DIETHOFENCARB | 87130-20-9 |
| DIETHYL DITHIOBIS | 502-55-6 |
| DIETHYLTOLUAMID | 134-62-3 |
| DIFENOCONAZOLE | 119446-68-3 |
| DIFLUBENZURON | 35367-38-5 |
| DIFLUFENICAN | 83164-33-4 |
| DIFLUFENSOPYR | 109293-97-2 |
| DIFLUMETORIM | 130339-07-0 |
| DIMETHENAMID | 87674-68-8 |
| DIMETHENAMID-P | 163515-14-8 |
| DIMETHIPIN | 55290-64-7 |
| DIMETHIRIMOL | 5221-53-4 |
| DIMETHOATE | 60-51-5 |
| DIMETHOMORPH | 110488-70-5 |
| DIMOXYSTROBIN | 145451-07-6 |
| DINEX | 317-83-9 |
| DINICONAZOL | 76714-88-0 |
| DINITRAMINE | 29091-05-2 |
| DINITRAMINE | 29091-05-2 |
| DINOTEFURAN | 165252-70-0 |
| DIPHENAMID | 957-51-7 |
| DIPHENYL | 92-52-4 |
| DIPHENYLAMINE | 122-39-4 |
| DIPROGULIC ACID | 18467-77-1 |
| DIPYRITHIONE | 3696-28-4 |
| DIQUAT DIBROMIDE | 85-00-7 |
| DIRAX | 86-88-4 |
| DISULFIRAM | 97-77-8 |
| DITHIANON | 3347-22-6 |
| DITHIOPYR | 97886-45-8 |
| DIURON | 330-54-1 |
| DNOC | 534-52-1 |
| DODEMORPH | 1593-77-7 |
| DODINE | 2439-10-3 |
| DYMRON | 42609-52-9 |
| Formularbeginn  E 0858 Formularende | 112860-04-5 |
| ENDAVEN | 22212-55-1 |
| ENDOTHAL | 145-73-3 |
| EPN | 2104-64-5 |
| EPOXYCONAZOLE | 135319-73-2 |
| ETACONAZOLE | 60207-93-4 |
| ETAFOS | 38527-91-2 |
| ETHABOXAM | 162650-77-3 |
| ETHALFLURALIN | 55283-68-6 |
| ETHAMETSULFURON-METHYL | 97780-06-8 |
| ETHEPON | 16672-87-0 |
| ETHIOLATE | 2941-55-1 |
| ETHIPROLE | 181587-01-9 |
| ETHIRIMOL | 23947-60-6 |
| ETHOFENPROX | 80844-07-1 |
| ETHOPROPHOS | 13194-48-4 |
| ETHOXIQUIN | 91-53-2 |
| ETHOXYSULFURON | 126801-58-9 |
| ETOBENZANID | 79540-50-4 |
| ETOXIFEN | 131086-42-5 |
| ETRIDIAZOLE | 2593-15-9 |
| FALIMORPH | 1704-28-5 |
| FAMOXADONE | 131807-57-3 |
| FENAMIDONE | 161326-34-7 |
| FENAMIDONE | 161326-34-7 |
| FENAMINOSULF | 140-56-7 |
| FENAPANIL | 61019-78-1 |
| FENARIMOL | 60168-88-9 |
| FENASULAM | 78357-48-9 |
| FENAZACHIN | 120928-09-8 |
| FENBUCONAZOLE | 114369-43-6 |
| FENCHLORAZOLE | 103112-36-3 |
| FENCHLORAZOLE | 103112-36-3 |
| FENCHLORAZOLE-ETHYL | 103112-35-2 |
| FENCLORIM | 3740-92-9 |
| FENETHCARB | 30087-47-9 |
| FENFURAM | 24691-80-3 |
| FENHEXAMID | 126833-17-8 |
| FENITROPAN | 77834-86-7 |
| FENOXANIL | 115852-48-7 |
| FENOXAPROP | 95617-09-7 |
| FENOXAPROP-ETHYL | 66441-23-4 |
| FENOXAPROP-ETHYL | 95617-09-7 |
| FENOXYCARB | 72490-01-8 |
| FENPICLONIL | 74738-17-3 |
| FENPROPATHRIN | 39515-41-8 |
| FENPROPIDIN | 67306-00-7 |
| FENPROPIDIN | 67306-00-7 |
| FENPROPIMORPH, (Z/E)- | 67306-03-0 |
| FENPYRAD | 119168-77-3 |
| FENPYROXIMATE | 111812-58-9 |
| FENTHIAPROP-ETHYL | 66441-11-0 |
| FENTIN HYDROXIDE | 76-87-9 |
| FENTIN-ACETAT | 900-95-8 |
| FENTRAZAMIDE | 158237-07-1 |
| FENURON | 101-42-8 |
| FENVALERATE | 51630-58-1 |
| FERIMZONE | 89269–64–7 |
| FIPRONIL | 120068-37-3 |
| FLAMPROP-ISOPROPYL | 57973-67-8 |
| FLAMPROP-METHYL | 52756-25-9 |
| FLAMPROP-M-ISOPROPYL | 63782-90-1 |
| FLAZASULFURON | 104040-78-0 |
| FLOCOUMAFEN | 90035-08-8 |
| FLONICAMID | 158062-67-0 |
| FLUACRYPYRIM 20C | 178813-81-5 |
| FLUAZIFOP | 69335-91-7 |
| FLUAZIFOP-BUTYL | 69806-50-4 |
| FLUAZINAM | 79622-59-6 |
| FLUBENDIAMIDE | 272451-65-7 |
| FLUBENZIMINE | 37893-02-0 |
| FLUCARBAZONE | 181274-17-9 |
| FLUCHLORALIN | 33245-39-5 |
| FLUCYTHRINATE | 70124-77-5 |
| FLUDIOXANIL | 131341-86-1 |
| FLUFENACET | 142459-58-3 |
| FLUFENERIM | 170015-32-4 |
| FLUFENOXURON | 101463-69-8 |
| FLUMETHRIN | 69770-45-2 |
| FLUMETOVER | Formularbeginn  154025-04-4 Formularende |
| FLUMETSULAM | 98967-40-9 |
| FLUMIOXAZIN | 103361-09-7 |
| FLUMORPH | 211867–47–9 |
| FLUOBUTRACIL | 134605-64-4 |
| FLUOMETURON | 2164-17-2 |
| FLUOPICOLIDE | 239110-15-7 |
| FLUORODIFEN | 15457-05-3 |
| FLUOROGLYCOFEN-ETHYL | 77501-90-7 |
| FLUOROIMIDE | 41205-21-4 |
| FLUOTRIMAZOLE | 31251-03-3 |
| FLUPROPAZIL | 120890-70-2 |
| FLUPYRSULFURON-METHYL-SODIUM | 144740-54-5 |
| FLUQINCONAZOLE | 136426-54-5 |
| FLURAZOLE | 72850-64-7 |
| FLURECOL-BUTYL | 2314-09-2 |
| FLURENOL | 467-69-6 |
| FLURIDONE | 59756-60-4 |
| FLUROCHLORIDONE | 61213-25-0 |
| FLURTAMONE | 96525-23-4 |
| FLUSILAZOLE | 85509-19-9 |
| FLUTOLANIL | 66332-96-5 |
| FLUTRIAFOL | 76674-21-0 |
| FLUTRIAFOL | 76674-21-0 |
| FLUVALINAT | 69409-94-5 |
| FLUXAPYROXAD | 907204-31-3 |
| FOLPAN | 133-07-3 |
| FOMESAFEN | 72178-02-0 |
| FONOFOS | 944-22-9 |
| FORAMSULFURON | 173159-57-4 |
| FORCHLORFENURON | 68157-60-8 |
| FORMETANATE | 23422-53-9 |
| FOSAMINE-AMMONIUM | 25954-13-6 |
| FOSAMINE-AMMONIUM | 59682-52-9 |
| FTHALIDE | 27355–22–2 |
| FUBERIDAZOLE | 3878-19-1 |
| FURALAXYL | 57646-30-7 |
| FURCARBANIL | 28562-70-1 |
| FURCONAZOL | 112839-33-5 |
| FURCONAZOL | 112839-33-5 |
| FURILAZOLE | 121776-33-8 |
| FURMETAMID | 60568-05-0 |
| GIBBERELLIC ACID | 77-06-5 |
| GLIOTOXIN | 67-99-2 |
| GLUFOSINATE | 51276-47-2 |
| GLYPHOSATE | 1071-83-6 |
| GLYPHOSINE | 2439-99-8 |
| GRISEOFULVIN | 126-07-8 |
| GUAZATINETRIACETATE | 39202-40-9 |
| HALACRINATE | 34462-96-9 |
| HALOXYFOP | 69806-34-4 |
| HALOXYFOP-METHYL | 69806-40-2 |
| HEPTOPARGIL | 73886-28-9 |
| HEXACHLORCYCLOHEXAN | 58-89-9 |
| HEXACHLOROFEN | 70-30-4 |
| HEXACONAZOL | 79983-71-4 |
| HEXADECYL CYCLOPRPANECARBOXYLATE | 54460-46-7 |
| HEXAFLUMURON | 86479-06-3 |
| HEXAZINONE | 51235-04-2 |
| HGW86 | 736994-63-1 |
| HYDRAMETHYLNON | 89269-64-7 |
| HYDRAMETHYLNON | 67485-29-4 |
| IMAZALIL | 35554-44-0 |
| IMAZAMOX | 114311-32-9 |
| IMAZAPIC | 104098-48-8 |
| IMAZAPYR | 81334-34-1 |
| IMAZAPYR-ISOPROPYLAMIN | 81510-83-0 |
| IMAZAQUIN | 81335-37-7 |
| IMAZETHABENZ | 81405-85-8 |
| IMAZETHAPYR | 81335-77-5 |
| IMAZOSULFURON | 122548-33-8 |
| IMIDACLOPRID | 138261-41-3 |
| INABENFIDE | 82211-24-3 |
| INDOXACARB | 144171-61-9 |
| IODOSULFURON-METHYL-SODIUM | 144550-36-7 |
| IOXINYL | 1689-83-4 |
| IPCONAZOLE | 125225–28–7 |
| IPROBENFOS | 26087-47-8 |
| IPRODIONE | 36734-19-7 |
| ISOFENPHOS | 25311-71-1 |
| ISOPROCARB | 2631-40-5 |
| ISOPROPALIN | 33820-53-0 |
| ISOPROTHIOLANE | 50512-35-1 |
| ISOPROTURON | 34123-59-6 |
| ISOPYRAZAM | 881685-58-1 |
| ISOURON | 55861-78-4 |
| ISOXABEN | 82558-50-7 |
| ISOXACHLORTOLE | 141112–06–3 |
| ISOXADIFEN DIFENOXALINE | 209866-92-2 |
| ISOXADIFEN-ETHYL | 163520-33-0 |
| ISOXAFLUTOLE | 141112-29-0 |
| KARANJIN | 521-88-0 |
| KETOSPIRADOX | 187270-87-7 |
| KINETIN | 525-79-1 |
| KRESOXIM-METHYL | 143390-89-0 |
| LACTOFEN | 77501-63-4 |
| LENACIL | 2164-08-1 |
| LINURON | 330-55-2 |
| LUFENURON | 103055-07-8 |
| MALATHION | 121-75-5 |
| MALEIC HYDRAZIDE | 123-33-1 |
| MANDIPROPAMID | 374726-62-2 |
| MANEB | 12427-38-2 |
| MCPA-ETHYL | 2698-38-6 |
| MCPA-METHYLESTER | 2436-73-9 |
| MCPB | 94-81-5 |
| MDPC | 25217-43-0 |
| MECOPROP | 93-65-2 |
| MECOPROP-METHYLESTER | 2786-19-8 |
| MEFENACET | 73250-68-7 |
| MEFENPYR-DIETHYL | 135590-91-9 |
| MEPANIPYRIM | 110235-47-7 |
| MEPIQUAT CHLORIDE | 24307-26-4 |
| MEPRONIL | 55814-41-0 |
| MESOTRIONE | 104206-82-8 |
| METAFLUMIZONE | 139968-49-3 |
| METALAXYL | 57837-19-1 |
| METAMIFOP | 256412-89-2 |
| METAMITRON | 41394-05-2 |
| METAZACHLOR | 67129-08-2 |
| METCONAZOLE | 125116-23-6 |
| METHABENZTHIAZURON | 18691-97-9 |
| METHAMIDOPHOS | 10265-92-6 |
| METHAZOLE | 20354-26-1 |
| METHFUROXAM | 28730-17-8 |
| METHIDATHION | 950-37-8 |
| METHOPREN | 40596-69-8 |
| METHOXYCHLOR | 72-43-5 |
| METHYL ANTHRANILATE | 134-20-3 |
| METHYLNEODECANAMIDE | 105726-67-8 |
| METOBROMURON | 3060-89-7 |
| METOLACHLOR | 51218-45-2 |
| METOLACHLOR | 51218-45-2 |
| METOLCARB | 1129-41-5 |
| METOMINOSTROBIN | 133408-50-1 |
| METOXURON | 19937-59-8 |
| METRAFENONE | 220899-03-6 |
| METSULFOVAX | 21452-18-6 |
| METSULFURON-METHYL | 74223-64-6 |
| MILDIOMYCIN | 67527-71-3 |
| MOLINATE | 2212-67-1 |
| MON-4660 | 71526-07-3 |
| MONOLINURON | 1746-81-2 |
| MYCLOBUTANIL | 88671-89-0 |
| MYCLOZOLIN | 54864-61-8 |
| NAPROPAMID | 15299-99-7 |
| NAPTALAM | 132-66-1 |
| NEBURON | 555-37-3 |
| NICLOSAMIDE | 1420-04-8 |
| NICOTINE | 54-11-5 |
| NITHIAZINE | 58842-20-9 |
| NITRALIN | 4726-14-1 |
| NITRAPYRIN | 1929-82-4 |
| NITROFEN | 1836-75-5 |
| NITROTHAL-ISOPROPYL | 10552-74-6 |
| NORFLURAZON | 27314-13-2 |
| N-TRITYLMORPHOLINE | 1420-06-0 |
| NUARIMOL | 63284-71-9 |
| OFURACE | 58810-48-3 |
| O-PHENYLPHENOL | 132-27-4 |
| ORYSASTROBIN | 248593-16-0 |
| ORYZALIN | 19044-88-3 |
| OSTHOLE | 484-12-8 |
| OVEX | 80-33-1 |
| OXABENTRANIL | 74782-23-3 |
| OXACICLOMEFONE | 153197-14-9 |
| OXADIAZON | 19666-30-9 |
| OXADIXYL | 77732-09-3 |
| OXAMYL | 23135-22-0 |
| OXASULFURON | 144651-06-9 |
| OXIFLUORFEN | 42874-03-3 |
| OXIPURINOL | 4318-51-8 |
| OXOLINIC ACID | 14698-29-4 |
| PACLOBUTRAZOL | 66346-04-1 |
| PACLOBUTRAZOL | 66346-04-1 |
| PARAQUAT CHLORIDE | 1910-42-5 |
| PARASULFON | 80-00-2 |
| PARATHION | 56-38-2 |
| PARATHIONMETHYL | 298-00-0 |
| P-CHLOROPHENOXYACETIC ACID | 122-88-3 |
| PEBULATE | 1114-71-2 |
| PELARGONIC ACID | 112-05-0 |
| PENCONACOL | 66246-88-6 |
| PENCYCURON | 66063-05-6 |
| PENDIMETHALIN | 40487-42-1 |
| PENFLUFEN | 494793-67-8 |
| PENTHIOPYRAD | 183675-82-3 |
| PERFLURON | 35367-31-8 |
| PERMETHRIN | 51877-74-8 |
| PETHOXYAMID | 106700-29-2 |
| PHENAZIN-N-OXID | 304-81-4 |
| PHENOTHIOL | 25319-90-8 |
| PHENOTHRIN | 26002-80-2 |
| PHENYL SULFONE | 127-63-9 |
| PHORATE | 298-02-2 |
| PHOSPHOCARB | 126069-54-3 |
| PHOSPHOLANE | 947-02-4 |
| PHOSPHON D | 115-78-6 |
| PHOXIM-METHYL | 14816-16-1 |
| PHTHALANILIC ACID | 4727-29-1 |
| PICLORAM | 1918-02-1 |
| PICOLINAFEN | 137641-05-5 |
| PICOXYSTROBIN | 117428–22–5 |
| PINOXADEN | 243973-20-8 |
| PIPERALIN | 3478-94-2 |
| PIPERONYL BUTOXIDE | 51-03-6 |
| PIPEROPHOS | 24151-93-7 |
| PRECOCEN II | 644-06-4 |
| PRECOCENE | 65383-73-5 |
| PRETILACHLOR | 51218-49-6 |
| PRIMISULFURON-METHYL | 86209-51-0 |
| PROBENAZOLE | 27605-76-1 |
| PROCARBAZONE | 145026-81-9 |
| PROCHLORAZ | 67747-09-5 |
| PROCYMIDONE | 32809-16-8 |
| PRODIAMINE | 29091-21-2 |
| PROFENFOS | 41198-08-7 |
| PROFLURALIN | 26399-36-0 |
| PROHEXADION | 88805-35-0 |
| PROMETON | 1610-18-0 |
| PROPACHLOR | 1918-16-7 |
| PROPANIL | 709-98-8 |
| PROPARGITE | 2312-35-8 |
| PROPICONAZOL | 60207-90-1 |
| PROPOXUR | 114-26-1 |
| PROPYZAMID | 23950-58-5 |
| PROQUINAZID | 189278-12-4 |
| PROSULFURON | 94125-34-5 |
| PROTHIOCONAZOLE | 178928–70–6 |
| PROTHIOFOS | 34643-46-4 |
| PYMETROZINE | 123312-89-0 |
| PYRACARBOLID | 24691-76-7 |
| PYRACLOSTROBIN | 175013-18-0 |
| PYRAMETOSTROBIN | 915410-70-7 |
| PYRAOXYSTROBIN | 862588–11–2 |
| PYRAOXYSTROBIN | 862588-11-2 |
| PYRASULFOTOLE | 365400-11-9 |
| PYRAZOLATE | 58011-68-0 |
| PYRAZOPHOS | 13457-18-6 |
| PYRAZOSULFURON-ETHYL | 93697-74-6 |
| PYRIDABEN | 96489-71-3 |
| PYRIDAFOL | 40020-01-7 |
| PYRIDAPHENTHION | 7035-04-3 |
| PYRIDATE | 55512-33-9 |
| PYRIFENOX | 88283–41–4 |
| PYRIFENOX | 88283-41-4 |
| PYRIFLUQUINAZON | 337458-27-2 |
| PYRIMETHANIL | 53112-28-0 |
| PYRIMINIL | 53558-25-1 |
| PYRIPROXYFEN | 95737-68-1 |
| PYRITHIOBAC | 123342-93-8 |
| PYROQUILON | 57369-32-1 |
| PYROXASULFONE | 447399-55-5 |
| QUINCLORAC | 84087-01-4 |
| QUINMERAC | 90717-03-6 |
| QUINOFOP-ETHYL | 76578-14-8 |
| QUINOXYFEN | 124495-18-7 |
| QUINTOZEN | 82-68-8 |
| QUIZALOFOP | 76578-12-6 |
| QUIZALOFOP-ETHYL | 76578-14-8 |
| RESMETHRIN | 10453-86-8 |
| RH5849 | 112225-87-3 |
| RIMSULFURON | 122931-48-0 |
| ROTENONE | 83-79-4 |
| RYNAXYPYR | 500008-45-7 |
| SAFLUFENACIL | 372137-35-4 |
| SALICYLANILID | 87-17-2 |
| SANTONIN | 481-06-1 |
| SEDAXANE | 874967-67-6 |
| SERITARD | 82211-24-3 |
| SETHOXYDIM | 74051-80-2 |
| SIDURON | 1982-49-6 |
| SIMAZINE | 122-34-9 |
| SIRMATE | 1966-58-1 |
| SPIROXAMINE | 118134-30-8 |
| SULCOTRIONE | 99105-77-8 |
| SULFOMETHURONMETHYL | 74222-97-2 |
| SULFOTEP | 3689-24-5 |
| SULFOXAFLOR | 946578–00–3 |
| SULGLYCAPIN | 51068-60-1 |
| TCNB | 117-18-0 |
| TEBUCONAZOLE | 80443-41-0 |
| TEBUTAM | 35256-85-0 |
| TECHNICAL BROMACIL | 314-40-9 |
| TEDION | 116-29-0 |
| TEFLUBENZURON | 83121-18-0 |
| TEMBOTRIONE | 335104-84-2 |
| TEPRALOXYDIM | 149979-41-9 |
| TERBACIL | 5902-51-2 |
| TERBUFOS | 13071-79-9 |
| TERBUTRYN | 886-50-0 |
| TETCYCLACIS | 65245-23-0 |
| TETRACONAZOLE | 112281-77-3 |
| TETRAMETHRIN | 7696-12-0 |
| THENYLCHLOR | 96491-05-3 |
| THIABENDAZOLE | 148-79-8 |
| THIACLOPRID | 111988-49-9 |
| THIADIAZURON | 51707-55-2 |
| THIAMETURON-METHYL | 79277-27-3 |
| THIAZAFLURON | 25366-23-8 |
| THIFLUZAMIDE | 130000-40-7 |
| THIOCYCLAM | 31895-22-4 |
| THIOCYCLAM | 31895-22-4 |
| THIODICARB | 59669-26-0 |
| THIOLUTIN | 87-11-6 |
| THIOPHANATE-METHYL | 23564-05-8 |
| THIRAM | 137-26-8 |
| TOLCLOFOS-METHYL | 57018-04-9 |
| TOLFENPYRAD | 129558-76-5 |
| TOPRAMEZONE | 210631-68-8 |
| TRALKOXYDIM | 87820-88-0 |
| TRIADIMEFON | 43121-43-3 |
| TRIADIMENOL | 55219-65-3 |
| TRIAPENTHENOL | 76608-66-7 |
| TRIASULFURON | 82097-50-5 |
| TRIAZIFLAM | 131475–57–5 |
| TRIAZURON | 110895-43-7 |
| TRICHLOROACETIC ACID | 76-03-9 |
| TRICYCLAZOLE | 41814-78-2 |
| TRIDEMORPH | 24602-86-6 |
| TRIETAZINE | 1912-26-1 |
| TRIETHYLENEPHOSPHORAMIDE | 545-55-1 |
| TRIFLOXISTROBIN | 139485-98-6 |
| TRIFLUMIZOL | 68694-11-1 |
| TRIFLUMIZOL | 68694-11-1 |
| TRIFLURALIN | 1582-09-8 |
| TRIFLURON | 64628-44-0 |
| TRIFLUSULFURON-METHYL | 126535–15–7 |
| TRIFLUSULFURON-METHYL | 126535-15-7 |
| TRIFORINE | 26644-46-2 |
| TRINEXAPAC-ETHYL | 95266–40–3 |
| TRITICONAZOLE | 131983-72-7 |
| TRITICONAZOLE | 131983-72-7 |
| TRITOSULFURON | 142469-14-5 |
| UNICONAZOLE | 83657-22-1 |
| VAMIDOATE | 2275-23-2 |
| VANALATE | 1929-77-7 |
| VERBUTIN | 185676-84-0 |
| VINCLOZOLIN | 50471-44-8 |
| VINICONAZOLE | 77175-51-0 |
| Z9-DDA | 16974-11-1 |
| Z9-DODECENYLACETATE | 16974-11-1 |
| ZARILAMID | 84527-51-5 |
| ZOLAPROFOS | 63771-69-7 |
| ZOXAMIDE | 156052-68-5 |
